# Machine learning-based prediction of adverse pregnancy outcomes in antiphospholipid syndrome using pregnancy antibody levels

**DOI:** 10.3389/fphys.2025.1617796

**Published:** 2025-08-25

**Authors:** Wanqing Liu, Ju Huang, Jun Xiao, Shanling Yan

**Affiliations:** ^1^ Department of Obstetrics and Gynecology, Deyang People’s Hospital, Deyang, Sichuan, China; ^2^ Department of Ultrasound, Deyang People’s Hospital, Deyang, Sichuan, China

**Keywords:** antiphospholipid syndrome (APS), adverse pregnancy outcomes (APO), machine learning, aβ2GP1, XGBoost

## Abstract

**Background:**

Antiphospholipid syndrome (APS) is a major immune-related disorder that leads to adverse pregnancy outcomes (APO), including recurrent miscarriage, placental abruption, preterm birth, and fetal growth restriction. Antiphospholipid antibodies (aPLs), particularly anticardiolipin antibodies (aCL), anti-β2-glycoprotein I antibodies (aβ2GP1), and lupus anticoagulant (LA), are considered key biomarkers for APS and are closely associated with adverse pregnancy outcomes. This is a prospective observational cohort study to use machine learning model to predict adverse pregnancy outcomes in APS patients using early pregnancy aPL levels and clinical features.

**Methods:**

This prospective study began data collection and follow-up for APS patients undergoing pregnancy monitoring in January 2023, and all data collection and follow-up were completed by January 2025. The samples were divided into the APO group and non-APO group. Multivariable logistic regression and ridge regression were used to identify independent predictive factors for adverse pregnancy outcomes. Six machine learning models were developed: Light Gradient Boosting Machine (LGBM), CatBoost, Extreme Gradient Boosting (XGBoost), Logistic Regression (LR), Random Forest (RF), and Multi-Layer Perceptron (MLP). The performance of these models was evaluated using the area under the receiver operating characteristic curve (AUROC), accuracy, sensitivity, specificity, and F1 score. The best-performing model was further explained using Shapley Additive Explanations (SHAP) analysis. Additionally, decision curve analysis (DCA) was performed to assess the clinical utility of the models.

**Results:**

A total of 708 patients were included. Ridge regression analysis identified aβ2GP1, LA1/LA2, aCL, gestational week at termination, age at first miscarriage, age, BMI during pregnancy, use of medication, >3 adverse pregnancies, 1–2 adverse pregnancies, preeclampsia, and natural miscarriage as significant predictors. Among the six models, the XGBoost model performed the best for predicting adverse pregnancy outcomes (AUROC = 0.864). Decision curve analysis (DCA) further confirmed the superiority of the XGBoost model, and feature importance analysis revealed that aβ2GP1 levels were the most important variable among the 12 factors.

**Conclusion:**

This study demonstrated that the XGBoost model, integrating aPL levels and clinical features, offers an effective approach to predicting adverse pregnancy outcomes in APS patients. The model enables clinicians to quickly and accurately identify high-risk pregnancies, providing valuable support for personalized clinical interventions and treatments.

## Introduction

Antiphospholipid syndrome (APS) is a major immune-related disorder that leads to adverse pregnancy outcome (APO), including recurrent miscarriage, fetal growth restriction, preterm birth, and placental abruption (Petri, 2020; Sawale, 2024; Aluf et al., 2024). Previous studies have demonstrated a significant association between antiphospholipid antibodies (aPLs), particularly anticardiolipin antibodies (aCL), anti-β2-glycoprotein I antibodies (aβ2GP1), and lupus anticoagulant (LA), and significant association with these adverse outcomes (Michael et al., 2024; Zhang et al., 2024; Umeta et al., 2023). However, inconsistencies exist in the literature regarding the relationship between aPL levels and pregnancy outcomes, especially in terms of their predictive ability for subsequent pregnancy outcomes (Hu and Liu, 2024; Park et al., 2024). While some studies suggest that elevated aPL levels may serve as a key tool for early screening of high-risk pregnancies, the predictive value of these antibodies remains inadequately validated due to unstandardized detection methods and significant individual variability (Sayeeda et al., 2023). Additionally, most existing studies rely on traditional statistical methods to analyze aPL levels, which are limited in capturing the potential nonlinearities and interactions within high-dimensional clinical data (Yildirim et al., 2023; Huang et al., 2024). Thus, the true value of incorporating aPL levels into multi-dimensional predictive models has not been fully explored.

In recent years, machine learning techniques have shown significant potential in handling complex data and developing predictive models, by integrating various clinical variables and biomarkers, machine learning can effectively overcome the limitations of traditional statistical methods (Lee et al., 2024; Mohsen, 2024). However, the application of machine learning in the context of pregnancy aPL levels and adverse pregnancy outcomes remains limited, with the effectiveness and stability of such models still requiring further validation.

Therefore, this study aims to explore the correlation between pregnancy aPLs levels and adverse pregnancy outcomes using machine learning techniques. The study also seeks to develop a predictive model using machine learning algorithms to provide new theoretical insights and practical guidance for the timely diagnosis and personalized treatment of APS during pregnancy, ultimately aiming to reduce the incidence of adverse pregnancy outcomes.

## Materials and methods

This study protocol was reviewed and approved by the Ethics Committee of Deyang People’s Hospital (2022-04-083-K01) and adhered to the principles established in the Declaration of Helsinki. The informed consent was obtained from all participants. Data security protocols were established to ensure patient confidentiality through comprehensive de-identification procedures. Electronic health records were extracted and anonymized prior to analysis, with all personal identifiers removed in accordance with institutional privacy protection standards.

### Study subjects

Data were collected from pregnant women who underwent antiphospholipid antibody testing at Deyang People’s Hospital starting in January 2023, with all data collection and follow-up completed by January 2025. Inclusion criteria: (1) All patients met the APS criteria, which included one clinical criterion and one laboratory criterion (Chinese Medical Association Rheumatology Branch, 2011); 1) Clinical criteria included vascular embolism and morbid pregnancy. Vascular embolism refers to >1 arterial, venous or small vessel thrombosis in any organ or tissue. Morbid pregnancy includes more than one unexplained morphologically normal stillbirth at ≥10 weeks of gestation, or more than one morphologically normal premature birth due to severe eclampsia, preeclampsia or severe placental insufficiency before 34 weeks of gestation, or more than three unexplained spontaneous abortions at ≤10 weeks of gestation, and maternal anatomical and hormonal abnormalities and parental chromosomal abnormalities must be excluded; 2) Laboratory indicators include the presence of LA in plasma, or the detection of medium to high titers of IgG/IgM aCL or aβ2GPI in serum, at least twice, with an interval of at least 12 weeks; (2) Singleton pregnancy; (3) Complete clinical data: including but not limited to the patient’s basic information, such as age, weight, previous pregnancy history, medical history and laboratory test data of antiphospholipid antibodies; (4) Clear pregnancy outcomes: including whether recurrent miscarriage, fetal growth restriction, premature birth, placental abruption and other adverse pregnancy outcomes occur.

The exclusion criteria were as follows: (1) Patients with major comorbidities: including patients with severe diabetes, hypertension, liver and kidney damage during pregnancy, which seriously affect the outcome of pregnancy or the immune system; (2) Patients receiving immunosuppressive therapy; (3) Patients with other immune-related diseases, such as systemic lupus erythematosus, rheumatoid arthritis and other autoimmune diseases. All patients gave informed consent, and this study was approved by the Ethics Committee of our hospital.

In this study, the gestational age at termination of pregnancy refers to the outcome of the current pregnancy, specifically the gestational age at termination of pregnancy, whether due to miscarriage, premature birth, or other pregnancy-related problems. This variable is used to assess the time of occurrence of pregnancy outcomes and is recorded as a baseline indicator before any adverse pregnancy outcomes occur. Natural miscarriage refers to spontaneous miscarriage that occurs before 20 weeks of pregnancy, usually confirmed by clinical diagnosis and medical records. This variable includes not only spontaneous miscarriages during the current pregnancy, but also spontaneous miscarriages that occurred in previous pregnancies. Preeclampsia refers to preeclampsia that occurs during the current pregnancy, specifically confirmed by clinical diagnosis and medical records. This variable is a complication of the current pregnancy and usually affects the prognosis of pregnancy outcomes. Use of medication refer to drug treatments given to patients to control pregnancy complications, usually drug interventions when high risks are found during pregnancy. The selection of all predictor variables is based on data before the outcome event occurs to ensure the time relevance of the data and the accuracy of the prediction.

**FIGURE 1:**
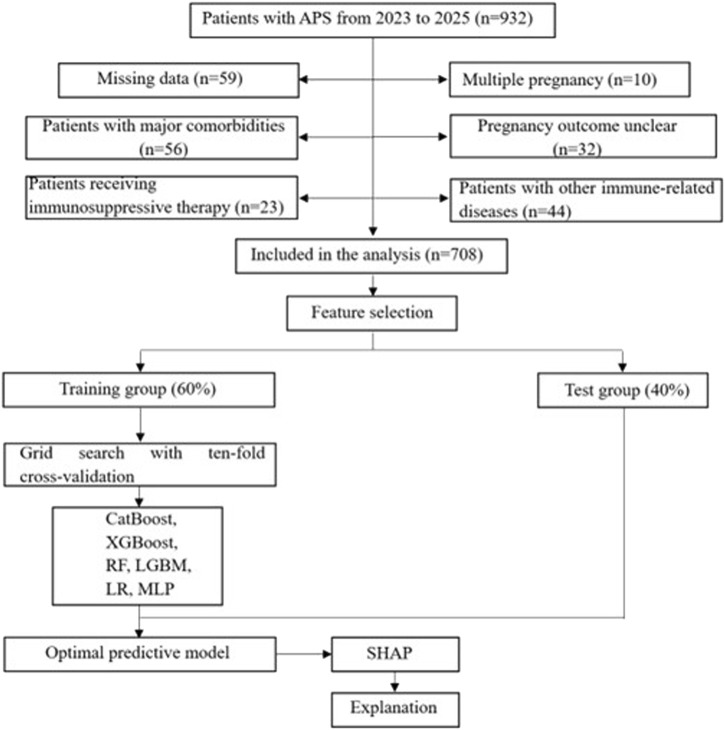
Flowchart of patient selection and machine learning model development process. LR, logistic regression; LGBM, light gradient boosting machine; CatBoost, categorical boosting; MLP, multilayer perceptron; RF, random forests; XGBoost, extreme gradient boost; SHAP, shape additive explanation.

After the positive aPLs were found, clinical drugs (LDA or LMWH) were given for intervention treatment, and antibodies were rechecked every 12 weeks until negative or delivery. Then they were divided into two groups according to whether APO occurred, with 322 patients in the APO group and 386 patients in the non-APO group. Figure 1 shows the flow chart of patient enrollment and study design. Finally, 708 APS patients were randomly divided into a training group (60%) and a test group (40%). The training group was used to train and optimize the machine learning models, while the test group was reserved to assess the predictive performance of these models. Importantly, feature selection and preprocessing were strictly confined to the training data to avoid any potential data leakage. In cases where data were missing for certain variables, we employed multiple imputation methods to handle missing data. Specifically, missing values for continuous variables were imputed using predictive mean matching, while missing categorical data were imputed using a multinomial logistic regression model.

### Research methods

2–3 mL of venous blood was collected from the subjects before 12 weeks of pregnancy in a fasting state and sent to the laboratory. The aCL and aβ2GP1 antibodies were detected by chemiluminescence immunoassay and tested in the Roche Cobas e411 chemiluminescence immunoassay instrument. This method quantitatively analyzes the antibody concentration through the specific binding reaction between antigen and antibody and the light signal generated by the chemiluminescence marker. LA1/LA2 was detected by the activated partial thromboplastin time test and tested in the sysmex 5100 automatic coagulation instrument.

### Observation indicators

The APS-related APOs consists of patients who experienced one or more APS-related adverse pregnancy outcomes. These outcomes include premature birth, miscarriage, stillbirth, preeclampsia, small for gestational age, premature rupture of membranes, and fetal distress. However, we excluded other potential causes of adverse pregnancy outcomes, such as anatomical abnormalities or cervical dilatation, that are not related to APS.

A patient was classified into the APO group if they had any one of these APS-related conditions, as confirmed by clinical diagnosis and medical records. This classification was applied regardless of the number or severity of the conditions, with the presence of any one of the aforementioned APS-related adverse pregnancy outcomes being sufficient for inclusion in the APO group.

(1) Premature birth: refers to birth at ≥28 weeks of gestation but <37 weeks of gestation.
(2) Miscarriage: refers to termination of gestation <28 weeks and fetal weight <1,000 g.
(3) Stillbirth: fetus dies *in utero* at gestation >20 weeks.
(4) Preeclampsia: systolic blood pressure ≥140 mmHg and/or diastolic blood pressure ≥90 mmHg after 20 weeks of gestation, accompanied by proteinuria ≥0.3 g/24 h, or random urine protein (+), or the absence of proteinuria but with one of the following: thrombocytopenia (platelets <100 × 109/L), liver function impairment (serum aminotransferase level is more than twice the normal value), renal function impairment (serum creatinine level >1.1 mg/dL or more than twice the normal value), pulmonary edema, new central nervous system abnormalities or visual impairment.
(5) Small for gestational age: a newborn whose birth weight is less than the 10th percentile for gestational age.
(6) Preterm premature rupture of membranes: spontaneous rupture of membranes before labor at ≥28 weeks but less than 37 weeks of gestation.
(7) Fetal distress: a combination of symptoms that endanger the health and life of the fetus due to acute or chronic hypoxia in the uterus.

### Development and validation of the predictive model

To ensure the simplicity of our model, we performed t-tests, Mann-Whitney U tests, and chi-square tests to select variables with statistically significant differences between the APO group and the non-APO group. Then, we applied 10-fold cross-validation with Ridge regression for feature selection and dimensionality reduction during model training. Additionally, grid search was used to optimize the hyperparameters of each model. To ensure consistent distribution of the outcome variable across all dimensions, we performed stratified sampling. Finally, we conducted multivariable logistic regression analysis on the non-zero coefficients to select independent risk factors and construct machine learning models. We developed six machine learning prediction models, including Light Gradient Boosting Machine (LGBM), Categorical Boosting (CatBoost), Extreme Gradient Boosting (XGBoost), Logistic Regression (LR), Random Forest (RF), and Multi-Layer Perceptron (MLP). The comprehensive hyperparameter configurations determined through systematic cross-validation are detailed in Supplementary Table S1. These optimized parameters were subsequently employed for final model training on the complete training dataset. The data was randomly divided into a training group (60%) and a testing group (40%). The training group was used for model development and hyperparameter tuning, while the testing group was used for model evaluation and validation. We used grid search with 10-fold cross-validation to find and determine the optimal parameters for the machine learning algorithms. The grid search algorithm systematically arranges and combines all possible parameter values, then substitutes the results of each combination into the model training process. The goal is to identify the optimal parameter combination from the exhaustive set of possibilities. The model’s predictive ability was assessed using discrimination and calibration validation. The area under the receiver operating characteristic curve (AUROC) represents a measure of discrimination, and model performance was evaluated based on accuracy, sensitivity, specificity, and F1 score. The Brier score and calibration curve were used for model calibration. The Brier score represents the mean squared deviation between predicted probabilities and actual labels. A lower Brier score indicates better model performance. The clinical utility and net benefit were assessed using Decision Curve Analysis (DCA). Shapley Additive Interpretation (SHAP) was used to directly elucidate the impact of important variables on the model. SHAP is a model explanation technique based on cooperative game theory, which has recently demonstrated its effectiveness in interpreting various machine learning models. Specifically, SHAP assigns a Shapley value to each feature by classifying the model’s output. Intuitively, estimating the Shapley value for each feature helps to explain its contribution to the outcome. The Shapley value accurately reflects the influence of each feature on a sample and aids in understanding whether it acts as a protective or risk factor within the model. The SHAP summary plot generated from the Shapley values ranks the importance of features, and SHAP force plots are used to analyze and interpret the prediction results for individual samples.

### Statistical analysis

All statistical analyses were performed using IBM SPSS (26.0), R (4.2.3), and Python (3.10.0). For continuous variables that followed a normal distribution, the data are presented as ( $\overset{–}{\text{x}}$ ± s), and comparisons between two groups were made using independent samples t-test. Categorical variables are presented as frequencies (%), and statistical analysis was performed using the *χ* ^*2*^ test or Fisher’s exact test. *P* < 0.05 was considered statistically significant.

## Results

### Clinical features and characteristics

A total of 708 APS pregnant patients were included in this study. The analysis comparing the APO group and the non-APO group revealed that the APO group had higher age, shorter gestational week at termination, lower age at first miscarriage, higher BMI during pregnancy, and a higher history of adverse pregnancy outcomes. After medication treatment, the number of APO patients significantly decreased. The APO group also had a higher risk of natural miscarriage and preeclampsia, along with higher antiphospholipid antibody levels. These differences were statistically significant (*P* < 0.05), as shown in Table 1.

**TABLE 1:** Clinical features and characteristics of all participants in different groups.

Clinical characteristics	Total (*n* = 708)	APO group (*n* = 322)	Non-APO group (*n* = 386)	t/X^2^ value	*p* value
Age (year)	31.25 ± 4.08	32.83 ± 3.24	30.26 ± 3.48	10.095	<0.001
Gestational week at termination (week)	35.65 ± 4.69	35.53 ± 4.62	36.68 ± 4.78	−3.236	0.001
Age at first miscarriage (year)	26.86 ± 4.12	25.58 ± 3.57	27.72 ± 4.82	−6.599	<0.001
BMI during pregnancy (kg/m^2^)	27.33 ± 5.62	27.65 ± 5.02	26.43 ± 3.73	3.716	<0.001
Diagnosis of APS disease duration (year)	2.69 ± 3.25	2.74 ± 3.10	2.63 ± 3.73	0.421	0.674
Past adverse pregnancy and childbirth history					
No adverse pregnancy history (%)	283 (40.0%)	127 (39.4%)	156 (40.4%)	0.069	0.792
1–2 adverse pregnancy (%)	326 (46.0%)	170 (57.1%)	156 (36.8%)	10.832	<0.001
>3 adverse pregnancy (%)	99 (14.0%)	62 (21.1%)	37 (8.0%)	13.646	0.005
Use of medication (%)	591 (83.5%)	251 (74.8%)	320 (90.7%)	13.066	<0.001
Pregnancy outcomes and obstetric complications					
Natural miscarriage (%)	62 (8.8%)	39 (12.1%)	23 (6.0%)	8.672	0.003
Fetal death (%)	25 (3.5%)	14 (4.3%)	11 (2.8%)	1.123	0.289
Live birth (%)	621 (87.7%)	290 (90.1%)	331 (85.8%)	3.072	0.082
Vaginal birth (%)	397 (56.1%)	188 (58.4%)	209 (54.1%)	1.281	0.258
Cesarean section (%)	224 (31.6%)	102 (31.7%)	122 (31.6%)	0.000	0.984
Preterm birth (<37 weeks) (%)	211 (34.0%)	103 (32.0%)	108 (28.0%)	1.452	0.228
Low birth weight (%)	25 (4.0%)	11 (3.4%)	14 (3.6%)	0.032	0.858
Preeclampsia (%)	62 (10.0%)	39 (12.1%)	23 (6.0%)	8.672	0.003
Premature rupture of membranes (%)	49 (7.9%)	23 (7.1%)	26 (6.7%)	0.052	0.82
Fetal distress (%)	74 (11.9%)	36 (11.2%)	38 (9.8%)	0.362	0.547
aCL (U/mL)	708 (100%)	51.32 ± 15.64	41.81 ± 12.26	9.07	<0.001
aβ2GP1 (U/mL)	708 (100%)	63.38 ± 17.21	42.66 ± 13.94	17.70	<0.001
LA1/LA2 (s)	708 (100%)	1.66 ± 1.31	1.01 ± 0.93	7.70	<0.001

### Feature selection

**FIGURE 2:**
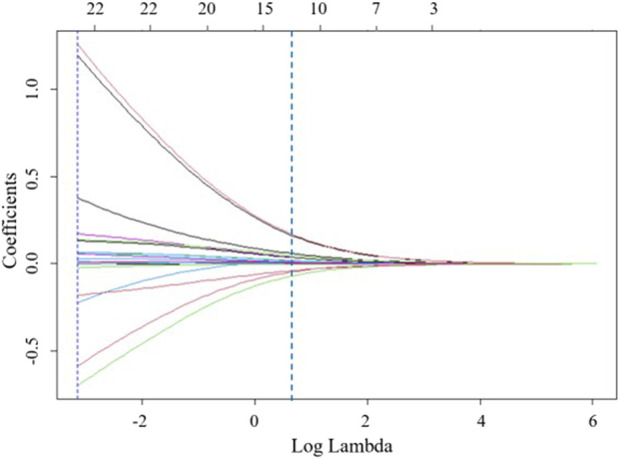
Feature selection based on ridge regression analysis, ridge regression coefficient profiles for 22 variables.

Ridge regression was used to prevent overfitting and address severe multicollinearity by shrinking the coefficients of variables. We performed ridge regression analysis and applied 10-fold cross-validation to filter variables. A lambda value with one standard error was used to select 12 variables (Figure 2), including age, gestational week at termination, age at first miscarriage, BMI during pregnancy, history of adverse pregnancy outcomes, use of medication, occurrence of natural miscarriage, preeclampsia, and aPLs levels. To further control for potential confounding factors, these 12 independent variables were analyzed using multivariable logistic regression (Table 2). And we found that these 12 variables were independent risk factors for the occurrence of APO. Ultimately, age, gestational week at termination, age at first miscarriage, BMI during pregnancy, history of adverse pregnancy outcomes, use of medication, occurrence of natural miscarriage, preeclampsia, and aPLs levels were selected for inclusion in the machine learning model.

**TABLE 2:** Based on ridge regression coefficients and Lambda. 1se values, validated the effectiveness of each variable using multivariable logistic regression.

Variables	Ridge regression		Multivariable logistics regression	
	Coefficients	Lambda. 1se	Or (95% CI)	*P*
Age	0.378622	0.042977	1.284 (1.217–1.355)	<0.001
Gestational week at termination	−0.58783		0.945 (0.914–0.977)	0.001
Age at first miscarriage (years)	−0.69784		0.878 (0.844–0.913)	<0.001
BMI during pregnancy	−0.22346		1.074 (1.036–1.114)	<0.001
1–2 adverse pregnancy	0.171435		2.291 (1.693–3.100)	<0.001
>3 adverse pregnancy	0.13167		3.066 (1.947–4.829)	0.005
Use of medication	−0.18025		0.306 (0.200–0.468)	<0.001
Natural miscarriage	0.055331		2.175 (1.270–3.726)	0.004
Preeclampsia	0.066098		2.175 (1.270–3.726)	0.004
aCL	1.195624		1.344 (1.275–1.418)	<0.001
aβ2GP1	1.259452		1.383 (1.293–1.480)	<0.001
LA1/LA2	0.137948		2.150 (1.798–2.570)	<0.001

### Performance of machine learning models and model interpretability

The data were randomly divided into a training group (60%, *N* = 425) and a testing group (40%, *N* = 283). Most predictive variables between the training and testing groups did not show statistically significant differences. In the training group, there were 193 APO patients and 232 non-APO patients. In the testing group, there were 129 APO patients and 154 non-APO patients. The 12 variables selected after feature selection were used as predictive variables to construct different predictive models. The optimized models were validated using 10-fold cross-validation on the training dataset, and grid search algorithms were used to find the best parameters for the machine learning models. The predictive performance of the models was then validated using the testing cohort.

**FIGURE 3:**
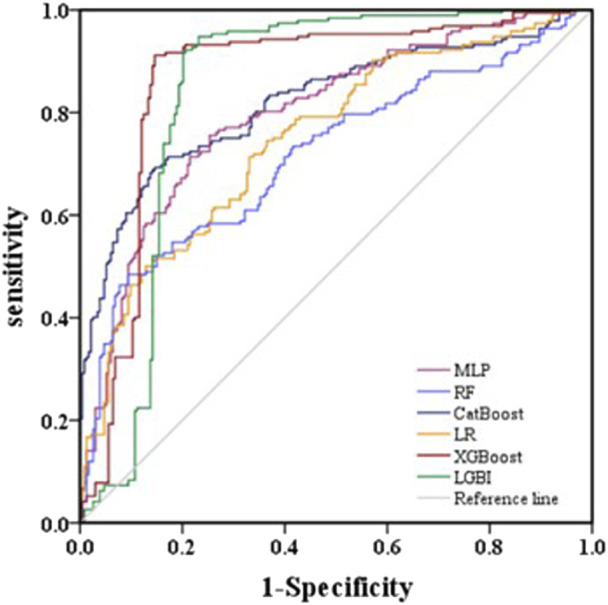
Evaluation of six machine learning models based on AUC of ROC curve in the validation set.

**FIGURE 4:**
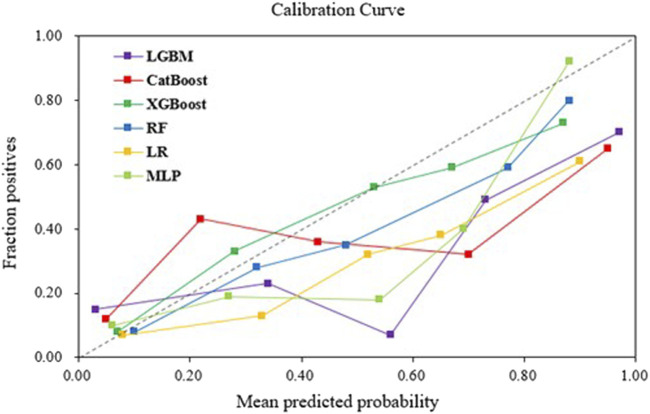
Calibration curves of machine learning models in the validation set.

**FIGURE 5:**
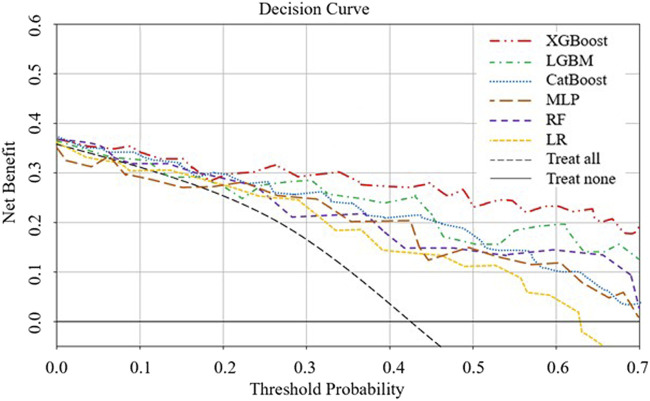
DCA analysis to evaluate clinical utility. The y-axis represents the net benefit, and the x-axis represents the threshold probability. Within a certain threshold range, the XGBoost model demonstrates higher net benefit.

The AUROC, accuracy, sensitivity, specificity, F1 score, and Brier score of the testing set are shown in Table 3. From the overall performance of each model, in terms of discrimination, the XGBoost model had the highest AUROC at 0.864, followed by the LGBM model (AUROC = 0.841), the CatBoost model (AUROC = 0.821), the MLP model (AUROC = 0.797), the LR model (AUROC = 0.749), and the RF model (AUROC = 0.718) (Figure 3). The corresponding Brier scores were 0.151, 0.117, 0.122, 0.121, 0.158, and 0.172, as shown in Figure 4. Decision Curve Analysis (DCA) demonstrated that the XGBoost model exhibited better clinical performance compared to the other models (Figure 5). Based on this comprehensive comparison, the XGBoost algorithm was selected to construct the predictive model.

**TABLE 3:** Performance of predictive models generated by machine learning models.

Model	AUROC (95% CI)	Accuracy (95% CI)	Sensitivity (95% CI)	Specificity (95% CI)	F1 score (95% CI)	Brier score (95% CI)
MLP	0.797 (0.755–0.817)	0.737 (0.707–0.753)	0.876 (0.859–0.890)	0.568 (0.544–0.587)	0.801 (0.784–0.823)	0.132 (0.118–0.156)
RF	0.718 (0.668–0.738)	0.680 (0.659–0.701)	0.768 (0.747–0.785)	0.573 (0.553–0.599)	0.721 (0.701–0.745)	0.149 (0.124–0.167)
LR	0.749 (0.702–0.753)	0.680 (0.662–0.717)	0.775 (0.750–0.796)	0.563 (0.537–0.584)	0.725 (0.712–0.748)	0.151 (0.132–0.174)
CatBoost	0.821 (0.779–0.842)	0.775 (0.753–0.788)	0.880 (0.867–0.901)	0.646 (0.623–0.672)	0.824 (0.809–0.843)	0.220 (0.203–0.246)
LGBM	0.841 (0.799–0.867)	0.741 (0.719–0.765)	0.845 (0.824–0.865)	0.615 (0.594–0.635)	0.790 (0.772–0.816)	0.161 (0.147–0.184)
XGBoost	0.864 (0.825–0.884)	0.769 (0.743–0.786)	0.884 (0.858–0.899)	0.630 (0.612–0.657)	0.823 (0.801–0.845)	0.158 (0.133–0.179)

**FIGURE 6:**
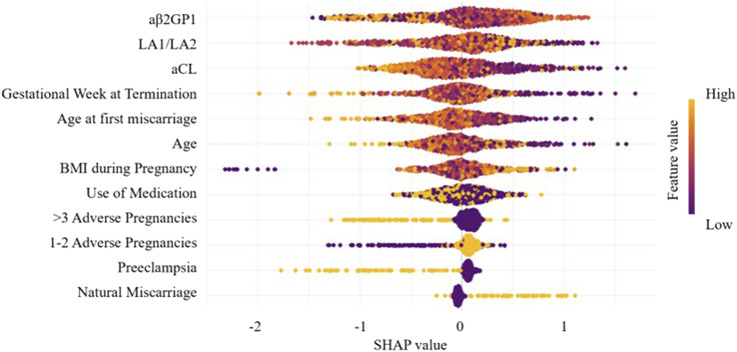
Feature importance SHAP summary plot. The scatter plot represents the contribution direction of each variable’s value, with yellow indicating higher values and purple indicating lower values.

**FIGURE 7:**
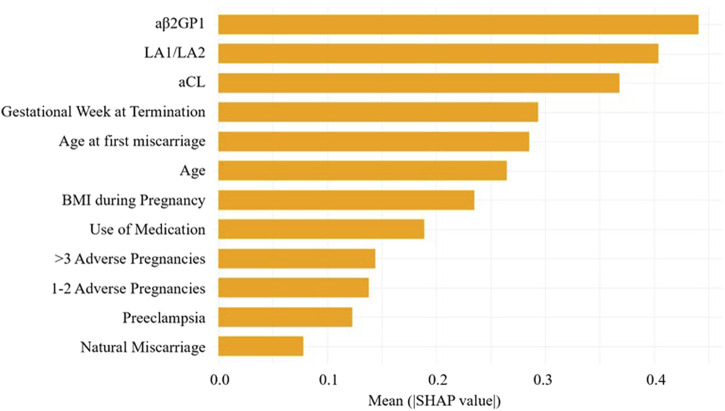
Feature importance SHAP bar chart. This chart displays the importance of each variable in the prediction model.

By calculating the contribution of each variable to the prediction, SHAP was used to interpret the results of the XGBoost model. The SHAP summary plot (Figure 6) is based on estimates, with each feature of every patient represented by a data point. Yellow represents higher values, while purple represents lower values. The horizontal axis shows the SHAP values, and larger shapes indicate features with higher predictive value for adverse pregnancy outcomes in a given sample. The importance bar chart (Figure 7) displays the significance of each variable in predicting adverse pregnancy outcomes. The variables are ranked in terms of their importance for predicting adverse pregnancy outcomes as follows: aβ2GP1, LA1/LA2, aCL, Gestational Week at Termination, Age at first miscarriage, Age, BMI during Pregnancy, Use of Medication, >3 Adverse Pregnancies, 1–2 Adverse Pregnancies, Preeclampsia, and Natural Miscarriage.

**FIGURE 8:**
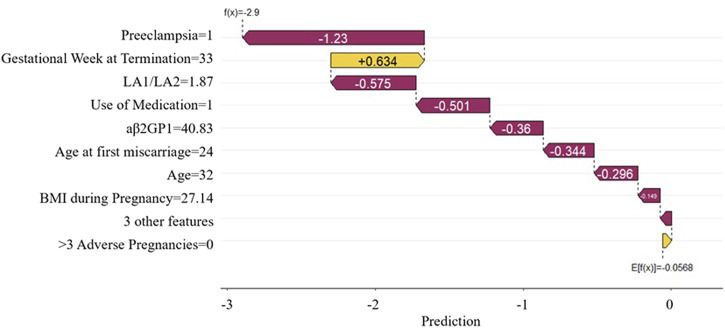
SHAP waterfall plot. This plot illustrates the contribution of each feature to the prediction result for a single sample.

The SHAP force plot applied to the predictive model effectively clarifies and explains the model’s predictions for individual patients. Figure 8 shows the SHAP waterfall plot of the XGBoost model, where the SHAP values represent the contribution of each relevant predictive feature for each patient, and how these features contribute to predicting adverse pregnancy outcomes. The length of the arrows helps to represent the magnitude of the prediction effect: the longer the arrow, the greater the effect. Figure 8 shows an APS patient with Preeclampsia and Use of Medication. The patient is currently 32 years old, with a Gestational Week at Termination of 33 weeks, LA1/LA2 of 1.87 s, aβ2GP1 of 40.83 U/mL, Age at first miscarriage of 24 years, and BMI during Pregnancy of 27.14 kg/m^2^. The Shapley value is −2.9 (<baseline value of −0.0568). The advantage of this force plot is that it clearly shows the parameter combinations that have the most significant contribution to the model.

## Discussion

This study explored the relationship between aPLs levels during pregnancy and APO patients, and constructed multiple machine learning models to predict these outcomes. The results showed that 12 variables, including aβ2GP1, LA1/LA2, aCL, Gestational Week at Termination, Age at first miscarriage, Age, BMI during Pregnancy, Use of Medication, >3 Adverse Pregnancy, 1–2 Adverse Pregnancy, Preeclampsia, and Natural Miscarriage, were significantly correlated with adverse pregnancy outcomes. Combining these key factors with machine learning models provides a theoretical basis for prediction and intervention of high-risk pregnancy individuals.

The role of aPLs in pregnancy outcomes has been widely studied, particularly their importance in predicting adverse pregnancy outcomes (Müller-Calleja et al., 2024). Previous studies have shown that elevated levels of aβ2GP1, aCL, LA1/LA2, may lead to immune system abnormalities that affect placental blood flow and function, thereby increasing the risk of fetal growth restriction, recurrent miscarriage, placental abruption, and other adverse pregnancy outcomes (Ren et al., 2024; Reshetnyak et al., 2023). While many studies have confirmed the correlation between antiphospholipid antibody levels and pregnancy complications, results across studies have been inconsistent (Murvai et al., 2025). Although the APS classification criteria require persistent medium to high titers of aPLs, the clinical significance of transient or low-titer positivity remains controversial (Mayer-Pickel et al., 2023). Arachchillage et al. (2015) found that intermittent or low-titer aPLs could also lead to sporadic reproductive failure, but women with occasional aPL positivity often do not present clinical symptoms of APS during their first pregnancy. This study further validates the importance of aCL, aβ2GP1, and LA1/LA2 antibodies in predicting adverse pregnancy outcomes. First, aβ2GP1 antibodies have been confirmed as one of the key variables in predicting adverse pregnancy outcomes, with elevated levels being closely associated with pregnancy complications such as fetal growth restriction, placental abruption, and pregnancy-induced hypertension (José and Tapia, 2023). Secondly, LA antibodies are closely related to the blood coagulation mechanism, and high levels of LA antibodies may affect placental blood flow and function, leading to adverse outcomes such as placental abruption, recurrent miscarriage, and fetal death (Horii et al., 2010). Therefore, the detection of LA antibodies is of great significance in identifying high-risk pregnant individuals, providing strong support for early intervention. Additionally, aCL antibodies, one of the most common antiphospholipid antibodies, have been confirmed to be closely associated with adverse pregnancy outcomes such as recurrent miscarriage, fetal distress, and preterm birth (Yun et al., 2023). In this study, the aCL antibody level was higher in the APO group, further emphasizing its importance as an effective biomarker for screening high-risk pregnant women. Thus, integrating APS antibody levels can provide an effective strategy for the early identification and intervention of high-risk pregnancies.

In the SHAP analysis, we identified several key features, such as aβ2GP1 levels and the history of adverse pregnancies, as having the most significant contributions to predicting APO. SHAP values revealed that higher aβ2GP1 levels were strongly associated with an increased risk of APO. Clinically, this means that elevated aβ2GP1 could serve as an early indicator for clinicians to monitor at-risk pregnancies more closely. By considering these features, clinicians can make more informed decisions about the need for early interventions or preventive measures. DCA was performed to assess the clinical utility of the model. Our analysis showed that the XGBoost model, when using a lower prediction threshold, improved the detection of high-risk pregnancies, including those with elevated aPL levels. This approach may lead to earlier interventions for high-risk individuals, even if the absolute risk is low, thereby preventing potential adverse pregnancy outcomes. Conversely, using a higher threshold for predicting APO could reduce unnecessary treatments for low-risk pregnancies, optimizing clinical resources. The flexibility in adjusting the threshold allows clinicians to tailor interventions based on individual patient risk profiles, further enhancing the model’s applicability in personalized medicine.

The study found that women with a history of adverse pregnancy outcomes are at higher risk in subsequent pregnancies, which is closely linked to elevated aPLs levels. Women with more than three previous adverse outcomes face an even greater risk. This highlights that a history of adverse pregnancy outcomes not only predicts future risks but also indicates potential immune system abnormalities associated with aPLs. Natural miscarriage was also a significant factor, with its higher incidence in the APO group linked to immune dysfunction caused by aPLs, which can disrupt placental development and increase miscarriage risk (Gharavi et al., 2010). These findings underscore the role of aPLs in predicting pregnancy complications, especially in women with a history of miscarriage. Elevated aPL levels indicate a higher risk of complications, suggesting that monitoring these levels and addressing immune abnormalities can provide preventive measures for high-risk pregnancies.

Preeclampsia is a common high-risk pregnancy disorder, closely linked to elevated antiphospholipid antibody levels and immune system abnormalities. Our study found that the proportion of preeclampsia patients in the APO group was significantly higher than in the non-APO group, suggesting a key role for antiphospholipid antibodies in the development of preeclampsia. Previous studies have shown that aβ2GP1 and aCL antibodies are strongly associated with preeclampsia, and reducing these antibody levels through treatment can significantly lower the risks of fetal growth restriction (FGR), intrauterine fetal distress, and preterm birth (Hanieh et al., 2019; Aline et al., 2010). Furthermore, our findings revealed that a higher proportion of patients in the APO group received medication, which raises an important consideration regarding potential confounding by indication. As medication use is typically prescribed to manage complex pregnancy complications, it is important to note that the decision to administer medication is based on clinical judgment and reflects a pre-existing assessment of higher pregnancy risk. This could introduce bias, as medication use may not be a direct predictor of adverse pregnancy outcomes, but rather a consequence of clinicians’ decisions to treat high-risk pregnancies. Therefore, while the use of medication is an important clinical intervention, it may also reflect the efficacy of treatment or clinicians’ *a priori* risk assessment, rather than serving as a baseline predictor of adverse pregnancy outcomes. This potential confounding effect must be taken into account when interpreting the results of our model.

Patients in the APO group were older and had a lower age at first miscarriage. As maternal age increases, the immune system may face more challenges, including elevated antiphospholipid antibody levels, which could significantly contribute to adverse pregnancy outcomes. A lower age at first miscarriage often signals immune abnormalities, particularly those related to antiphospholipid antibodies, increasing the risk of adverse outcomes in subsequent pregnancies. Therefore, age and age at first miscarriage are crucial for predicting adverse pregnancy outcomes, necessitating closer monitoring of these high-risk patients. Additionally, gestational week at termination was significantly associated with adverse pregnancy outcomes. A lower gestational week is linked to complications such as preterm birth and fetal distress, potentially influenced by elevated antiphospholipid antibody levels. Studies show that patients with earlier gestational termination are typically high-risk, with immune abnormalities affecting placental function and blood circulation. Such patients require enhanced monitoring and early intervention to reduce the risk of adverse outcomes. Furthermore, the BMI of APO group patients was significantly higher than that of the non-APO group. Higher BMI is associated with complications like gestational diabetes and hypertension, which increase the risk of fetal growth restriction through mechanisms such as thrombosis and impaired placental blood flow. High BMI not only correlates with elevated antiphospholipid antibody levels but may also impact pregnancy outcomes through multiple mechanisms. Therefore, maternal BMI, as a common risk factor, warrants close attention and appropriate monitoring and intervention in high-risk pregnancies.

Despite providing valuable theoretical support and practical guidance for the rapid screening and personalized treatment of APS during pregnancy, this study has several limitations. First, although the sample size includes 708 cases, it is derived from a single-center dataset, which may limit the external validity of the results. Second, while the current study provides promising results, the lack of external validation limits its generalizability. Future work should focus on validating the model in independent datasets to ensure robustness across different populations. And there is still room for improvement, particularly in enhancing the specificity and sensitivity of the model. Future research could attempt multi-center, large-scale data collection to further validate the model’s stability across different populations. Additionally, this study primarily relied on antiphospholipid antibody levels and basic clinical characteristics for prediction, without fully considering other potential biomarkers or genomic data, which could provide more comprehensive information for predicting pregnancy outcomes.

In conclusion, this study developed a machine learning model to predict APOs based on antiphospholipid antibody levels and clinical characteristics. The integration of SHAP values and DCA provides a comprehensive approach to understanding how different features and thresholds influence clinical decision-making. SHAP values identify the most significant risk factors, such as elevated aβ2GP1 levels, that are crucial for predicting APOs, allowing clinicians to focus on the most important indicators. DCA, on the other hand, helps determine the optimal threshold for clinical decisions, enabling clinicians to balance sensitivity and specificity. By adjusting the threshold, clinicians can ensure early identification and treatment of high-risk pregnancies, while avoiding unnecessary interventions for low-risk patients. The XGBoost model demonstrated the best performance, with high accuracy and clinical utility, making it adaptable to various clinical settings. Future research should focus on multi-center validation, dataset integration, and further clinical application to enhance the performance of the model, providing more effective screening and personalized interventions for high-risk pregnancies.

## Data Availability

The original contributions presented in the study are included in the article/Supplementary Material, further inquiries can be directed to the corresponding author.
