# Supplementary material for: Machine learning-based prediction of adverse pregnancy outcomes in antiphospholipid syndrome using pregnancy antibody levels
Source: Front Physiol. 2025 Aug 25;16:1617796. doi: 10.3389/fphys.2025.1617796 (PMC12415049; doi:10.3389/fphys.2025.1617796)
Supplement: Supplementary file 1 [file Table1.docx]

**Supplementary**

Table S1. Optimized hyperparameter configurations for the 6 algorithms

| Model | Parameter | Values |
| --- | --- | --- |
| MLP | Activation | ReLU |
|  | Solver | adam |
|  | Learning Rate | 0.001 |
|  | Max Iterations | 200 |
|  | Alpha | 0.0001 |
|  | Momentum | 0.9 |
|  | Batch Size | 200 |
| RF | n_estimators | 228 |
|  | max_features | None |
|  | max_depth | 18 |
|  | min_samples_split | 13 |
|  | min_samples_leaf | 3 |
|  | min_impurity_decrease | 0.0004 |
| LR | C | 0.239 |
|  | Penalty | L1 |
|  | Solver | saga |
|  | Tolerance | 8.18E-05 |
|  | max_iter | 1409 |
| CatBoost | iterations | 500 |
|  | learning_rate | 0.1 |
|  | depth | 8 |
|  | l2_leaf_reg | 3 |
|  | subsample | 0.8 |
|  | border_count | 128 |
|  | thread_count | -1 |
|  | task_type | CPU |
| LGBM | n_estimators | 1000 |
|  | learning_rate | 0.1 |
|  | max_depth | 12 |
|  | num_leaves | 31 |
|  | subsample_for_bin | 200000 |
|  | colsample_bytree | 0.8 |
|  | reg_alpha | 0.1 |
|  | reg_lambda | 0.1 |
| XGBoost | n_estimators | 261 |
|  | learning_rate | 0.1324 |
|  | max_depth | 14 |
|  | min_child_weight | 10 |
|  | Gamma | 0.9768 |
|  | Subsample | 0.9934 |
|  | colsample_bytree | 0.8 |
|  | reg_alpha | 0.1 |

C: Regularization parameter, saga: Stochastic average gradient with acceleration, max_iter: maximum number of iterations, n_estimators: number of trees in ensemble models, min_samples_split = minimum samples required to split an internal node, min_samples_leaf: minimum samples required to be at a leaf node, min_impurity_decrease: threshold for early stopping in tree growth, Gamma: Minimum loss reduction
